# Editorial: Illuminating carotenoid synthesis and plastid transition in plants, volume II

**DOI:** 10.3389/fpls.2023.1185632

**Published:** 2023-04-18

**Authors:** Claudia Stange, Li Li, Maria Jesús Rodrigo

**Affiliations:** ^1^ Department of Biology, Faculty of Science, University of Chile, Las Palmeras, Santiago, Chile; ^2^ Robert W. Holley Center for Agriculture and Health, USDA-ARS, Cornell University, Ithaca, NY, United States; ^3^ Institute of Agrochemistry and Food Technology, Spanish National Research Council (CSIC), Paterna, Spain

**Keywords:** carotenoids, plastids, chlorophylls, supplemental lighting, metabolic engineering, phytoene synthase (PSY)

Carotenoids are critically important metabolites for plants and for human health. Therefore, carotenoid metabolism and regulation have been studied comprehensively during the last forty years. Carotenoids are produced and stored in plastids. Genomics, transcriptomics, proteomics, and metabolomics have allowed the identification of genes and cellular processes important for the accumulation of carotenoids in plastids. The article contributed by Li et al. reviews some recent studies using “omics” approaches to better understand how plastids impact and enhance carotenoid accumulation.

## Light, an environmental key factor for carotenoid synthesis

Carotenoid synthesis is tightly regulated by several endogenous factors (senescence, circadian clock, epigenetic mechanisms, ABA feedback) and environmental cues, such as light (Sun et al., 2018; Quian et al., 2021). Light is a key factor that stimulates both plastid development and carotenoid synthesis and affects fruit yields and quality. In greenhouses, supplemental lighting is generally used to produce high-quality fruits year-round. Among various light sources, light-emitting diodes (LEDs) have the advantage of controlling the spectrum of light sources. Jang et al. reported that natural light supplemented with blue LED interlighting (RB) or RB with far-red light (RBFR) produced higher carotenoid contents as well as higher fruit yields and qualities than natural light in red and yellow sweet peppers (*Capsicum annuum* L.). These results suggest that adequate light quality and intensity are important to improve yield and plastid development in fruits. Clearly, the best light spectrum varies among crops and it is necessary to establish for each species.

The major rate-limiting enzyme of carotenogenesis is phytoene synthase (PSY) which catalyzes the first committed step in the carotenoid biosynthesis pathway. The review article contributed by Zhou et al. describes the current knowledge about the biology of PSY, including the regulation of PSY in response to diverse developmental and environmental cues, such as light. Evidences on the functional evolution, the dynamic regulation at the transcriptional, post-transcriptional, and post-translational level, and the metabolic engineering of PSY were addressed. Some open questions such as the key amino acid residues that give high PSY activity or the potential interaction with other enzymes to efficiently channeling the carotenoid biosynthetic flux were also discussed.

## Green plastids not only produce carotenoids, but also chlorophylls

Carotenoids and chlorophylls are produced from the same precursor, geranylgeranyl diphosphate (GGPP). It has been extensively shown that both pathways are interconnected. When a carotenogenic gene, such as *DcLCYB1*, is overexpressed in green tissues, carotenoid and chlorophyll levels increased along the expression of chlorophyll synthase (*NtCHL*) and carotenoid biosynthesis (*NtLCYB*, *NtPSY1* and *NtPSY2*) pathway genes (Moreno et al., 2016). Jang et al. investigated the genetic correlation of chlorophyll and carotenoid biosynthesis during *Capsicum annuum* fruit ripening. The authors demonstrated that high carotenoid and chlorophyll contents in the dark-green immature fruit are associated with increasing expression of 1-deoxy-d-xylulose 5-phosphate synthase (*DXS*) required for chlorophyll and carotenoid synthesis, as well as the expression of GOLDEN2-like (*CaGLK2*) transcription factor affected both chlorophyll and carotenoid synthesis in chloroplasts.

## Strategies for enhancing carotenoid accumulation in plants

The search for strategies to increase the level of carotenoids in plants facilitates the enrichment of the nutritional levels of our foods. One strategy pointed out refers to the identification and optimization of the key amino acid residues in PSY which could facilitate the design of highly efficient PSY for the development of carotenoid enriched crops (Zhou et al.). Another strategy was proposed, which is *via* the genetic manipulation and the use of environmental factors, such as light and temperature (Li et al.).

Stra et al.provided an overview of recent advances in our understanding of carotenoid metabolism and the development of new synthetic approaches for the production of carotenoids in crops and microorganisms. The article highlights recent studies in identifying new enzymes and regulatory factors involved in carotenoid metabolism and discusses how these findings can be used to improve carotenoid production. The authors also discussed the application of genome editing technologies, which offer promising avenues for the development of new carotenoid-based products in food, pharmaceutical and cosmetic industries.

## Author contributions

CS wrote the manuscript, LL and MR carefully revised the manuscript. All authors contributed to the article and approved the submitted version.
